# Herbal Fennel Essential Oil Nanogel: Formulation, Characterization and Antibacterial Activity against *Staphylococcus aureus*

**DOI:** 10.3390/gels8110736

**Published:** 2022-11-12

**Authors:** Aftab Alam, Ahmed I. Foudah, Mohammad Ayman Salkini, Mohammad Raish, Jyotiram Sawale

**Affiliations:** 1 Department of Pharmacognosy, College of Pharmacy, Prince Sattam Bin Abdulaziz University, Al Kharj 11942, Saudi Arabia; 2 Department of Pharmaceutics, College of Pharmacy, King Saud University, Riyadh 11451, Saudi Arabia; 3 IES Institute of Pharmacy, IES University Campus, Kalkheda, Ratibad Main Road, Bhopal 462044, India

**Keywords:** antimicrobial, antibiotics, *staphylococcus aureus*, essential oils, PLGA, nanogel

## Abstract

Antimicrobial resistance (AMR) is one of the greatest threats to humanity in the world. Antibiotic-resistant bacteria spread easily in communities and hospitals. *Staphylococcus aureus* (*S. aureus*) is a serious human infectious agent with threatening broad-spectrum resistance to many commonly used antibiotics. To prevent the spread of pathogenic microorganisms, alternative strategies based on nature have been developed. Essential oils (EOs) are derived from numerous plant parts and have been described as antibacterial agents against S. aureus. Fennel essential oils were selected as antibacterial agents encapsulated in nanoparticles of polylactic acid and glycolic acid (PLGA). The optimum size of the formulation after loading with the active ingredient was 123.19 ± 6.1595 nm with a zeta potential of 0.051 ± 0.002 (23 ± 1.15 mV). The results of the encapsulation efficiency analysis showed high encapsulation of EOs, i.e., 66.4 ± 3.127. To obtain promising carrier materials for the delivery of fennel EOs, they were incorporated in the form of nanogels. The newly developed fennel oils in PLGANPs nanogels have good drug release and MIC against *S. aureus*. These results indicate the potential of this novel delivery system for antimicrobial therapy.

## 1. Introduction

Infectious diseases induced by bacteria, fungi or viruses continue to be recognised and are growing medical problems around the world. It is necessary to continue the development of new antimicrobial materials [1]. Antimicrobial resistance, often called AMR, is one of the dominant threats to humans worldwide. According to analyzes of AMR cases in 204 countries and regions worldwide, 1.27 million people died directly from AMR in 2019 [2,3,4,5]. A new analysis estimates that 10 million people will die from AMR each year by 2050 if a global solution is not found [6]. Antibiotics are freely available and misused not only in hospitals but also in agriculture and animals, which has facilitated and accelerated the development of bacterial resistance to conventional antibiotics [7].

However, antibiotic resistance is not the main obstacle to effective infection control. as antibiotic-resistant bacteria are easily spread in communities and hospitals. This can happen directly, when an infected person touches a healthy person, or indirectly, when a person touches a contaminated surface [8]. *Staphylococcus aureus* (*S. aureus*) is one of the most common bacteria causing infections, and its resistance to broad-spectrum antibiotics is quite concerning. Although medical care has improved, the Gram-positive strain of *S. aureus* that causes hospital and community integrated infections is still accountable for many illnesses and 20% to 30% of deaths [9]. Pathogenic staphylococci are immobile, spherical and gram-positive bacteria about 1 µm in diameter. These infections are caused by staphylococcal virulence factors and often occur as skin and soft tissue infections [10].

*S. aureus* is an important human pathogen that has developed a broad spectrum of antibiotic resistance, including β-lactams, resulting in alarming levels of resistance. Antibiotic resistance poses a serious threat to the prevention and treatment of infectious diseases. A similar interest exists in antibiotics that inhibit and delay the development of bacteria [11]. In recent years, researchers have developed natural alternatives to combat the spread of disease-causing microbes. However, the development of new antibiotic-containing treatments has slowed for several reasons [12,13]. Essential oils (EOs) are one of the most diverse classes of bioactive chemicals produced by plants, and they play an important role in the plant’s defense response to a variety of disorders, including microbial infections. In recent years, the efficacy of many essential oils as broad-spectrum antimicrobials has been recognized. EO is extracted from many plant parts, including flowers, stems, twigs, fruits, seeds, roots, bark and leaves [14]. As an antibacterial, antifungal, anticarcinogenic, antimutagenic, antioxidant, anti-inflammatory and antiviral agent, this aromatic essential oil is widely used in pharmaceutical, agricultural, hygienic and therapeutic products [15]. Therefore, to address this problem, new drugs are needed to treat infections that are resistant to a variety of drugs.

Recently, numerous research papers have reported the antibacterial effect of fennel oil (FEO) [16,17,18]. Fennel (*Foeniculum vulgare* L.) is a plant of the Apiaceae family. The plant has long been used as a medicinal plant and is widely used in India, Egypt and other countries for its bitter leaves and aromatic seeds [17]. Its potent and broad-spectrum antimicrobial activity has led to impressive reports in the medical literature. The multicomponent composition of EOs also results in multitarget activity against multidrug-resistant bacteria (MDR), which is a significant advantage over the single-target activity of conventional antibiotics. In addition, EOs have the greatest potential for the development of microbiological resistance [19]. However, high production costs and lengthy approval processes hinder the development of new antibacterial drugs, which is particularly problematic given the rapid emergence of new strains of bacteria that are resistant to existing treatments [20].

Another interesting antibacterial technique currently under detailed investigation is the use of EOs as nanoscale delivery systems. In recent years, innovative delivery methods for antibiotics have been increasingly used in the context of combating antimicrobial resistance [21,22,23]. Recent breakthroughs in nanotechnology have enabled the production of nanoparticles (NPs) with precise size and shape, contributing to the development of new antibacterial drugs [24]. For instance, Siyadatpanah et al. reported green synthesis of nanoliposomes containing *Bunium persicum* (*B. persicum*) and *Trachyspermum ammi* (*T. ammi*) essential oils against *Trichomonas vaginalis*. The essential oils of *B. persicum* and *T. ammi*, combined in nanoliposomes, have the potential to be an effective alternative to conventional treatments for Trichomonas infections [25]. In this case, nanoforming can protect essential oils from external influences and control the distribution and location of essential oils.

Poly(lactide-co-glycolide) nanoparticles (PLGA) are increasingly being used for biomedical applications such as drug delivery because they are biodegradable and biocompatible and their release can be controlled [26,27,28]. In light of this, the objective of this study was to prepare polymeric PLGA nanoparticles (PLGANPs) containing FEO and to analyze their morphological characteristics, particle size, and encapsulation efficacy. In addition, this FEO–PLGANP is incorporated into Carbopol gel, which allows easy delivery to diseased sites. In our research project, we chose transdermal drug delivery via Carbopol gel. It provides more permeability, biocompatibility, flexibility, viscosity, skin hydration, washability and longer contact time between the drugs and the absorbent skin surface [29]. The prepared FEO–PLGANPs gels were also evaluated for their rheological properties, in vitro release profile and antimicrobial activity. To our knowledge, the antimicrobial activity of fennel essential oil in PLGANPs/gels has not yet been investigated.

## 2. Results and Discussion

### 2.1. Physicochemical Characterization of Nanoparticles

#### 2.1.1. Shape, Particle Size, Polydispersity Index and Zeta Potential

**Figure 1:**
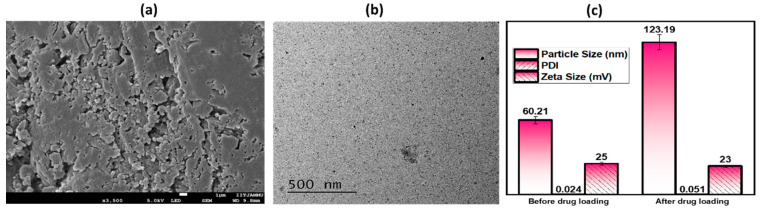
Physicochemical analysis: (**a**) FE-SEM image of gel prepared from FEO–PLGANPs; (**b**) TEM image of prepared gel; (**c**) measurements of particle size, polydispersity index and zeta potential of gel prepared from FEO–PLGANPs before and after drug loading.

The image obtained from FE-SEM is shown in Figure 1a. The microscopic FE-SEM image shows that the particles in the FEO–PLGANPs gel are distributed in the form of clusters with a variety of different shapes. However, in order to have a closer look, the morphology was further confirmed by TEM, as shown in Figure 1b. From the TEM, an irregular shape was observed. This may be due to the presence of gel in a wet state. The average particle size, PDI value and zeta potential of nanoparticles have a significant impact on the interaction of bioactive chemicals with target tissues in therapeutic drug delivery systems. The size of the particles before drug loading was 60.21 ± 3.0105 nm; the PDI was 0.024 ± 0.003; and the zeta potential was 25 ± 1.25 mV. The optimal size of the formulation after loading with the drug was 123.19 ± 6.1595 nm with a PDI of 0.051 ± 0.002 and a zeta potential of 23 ± 1.15 mV, as shown in Figure 1c. The results indicate that when FEOs are encapsulated with PLGANPs, their size increases somewhat and their zeta potential decreases proportionally. The sample showed exceptional particle homogeneity and had modest size distributions with low PDI values, indicating amazing particle homogeneity. It is possible to draw the conclusion that the ideal particle size of the formulation FEO–PLGANPs was satisfactory in that it was in excellent accord with the data that had been published in the past, showing that the population was homogenous [30]. When it comes to affecting bioavailability and shelf life, the surface charge of NPs remains an important factor [31]. In addition, the positive charge ensured the stability of the nanocomposite in the biological environment, which allowed access to the target sites [32].

#### 2.1.2. Drug Entrapment Efficiency (DEE%) of Nanoparticles

The results of the encapsulation efficiency analysis showed high encapsulation of the EO, i.e., 66.4 ± 3.127%. The results regarding the preparation of the FEO–PLGANPs showed that they were successfully prepared.

### 2.2. Characterization of FEO–PLGANPs Gel

#### 2.2.1. Rheological Study of FEO–PLGANPs Gel

Aiming at uniform dosage forms and excellent active ingredient carriers, we have developed a FEO–PLGANPs gel that can be applied directly to the skin surface. The gel containing FEO–PLGANPs had a pH of 6.0 ± 0.2, which is excellent for topical application and does not cause irritation. To test the spreadability of the developed gel, the viscosity was measured and reported as 36,176 cps. The optimal viscosity for topical preparation is in the range of 35,000 to 40,000 cps and provides both acceptable flow properties and spreadability [33]. In addition, the particle size and PDI value affect the viscosity of the gel. It was measured how far the FEO–PLGANPs gel could be spread, and the result was 8.76 ± 2.412 cm. After determining the viscosity of the gel, a satisfactory spreadability value was obtained. The excellent viscosity and spreadability of the topical gel allow easy application of a layer to the skin, as also reported in the literature [34].

#### 2.2.2. Drug Release Studies of FEO–PLGANPs Gel

**Figure 2:**
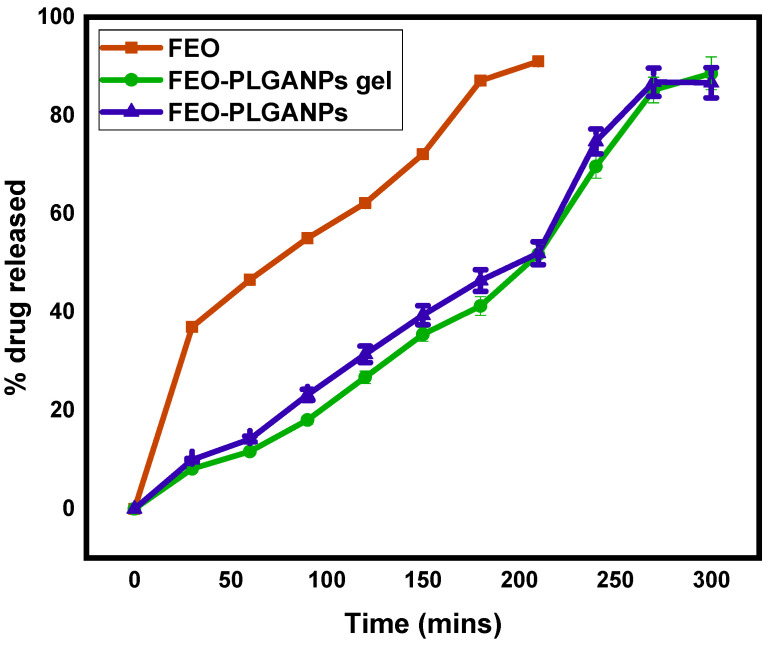
Comparative in vitro drug release study of FEO, FEO–PLGANPs and FEO–PLGANPs in a buffer solution of pH 6.8.

Figure 2 shows the comparative in vitro drug release profile of naked FEO, FEO–PLGANPs and FEO–PLGANPs gel (as topical gels). Then, the amount of drug released was measured in the selected media, i.e., buffer solution with pH 6.8. The amount of drug released varied depending on the formulation selected. In the in vitro drug release study, it was observed that more than 60% of naked FEO was released within 120 min, in contrast to FEO–PLGANPs gels (31.43%) and FEO–PLGANPs (26.76%). A total of 90% of the drug was released within 210 min in the case of FEO. The release of ug was maximal in the simulated intestinal fluid. The highest FEO release rate was found to be 88.55% for FEO–PLGANPs gels and 86.63% for FEO–PLGANPs in a buffer solution with pH 6.8 after 300 min. The drug release from topical gels was significantly higher than that of the corresponding FEO and FEO–PLGANPs.

Mathematical models of drug delivery systems play an essential role in their design, and understanding the consequences of the underlying processes and system parameters is critical. Experimental studies and modeling are currently underway to better understand the processes behind the kinetics of these release systems. Table 1 shows the drug release capacities and R^2^ values of bare FEO, FEO–PLGANPs and FEO–PLGANPs gels. In the case of FEO, the R^2^ value was reported as 0.9839, following Higuchi. For the topical gels FEO–PLGANPs and FEO–PLGANPs, R^2^ values of 0.996 and 0.9905, respectively, were reported, and both followed zero-order kinetics.

#### 2.2.3. In Vitro Cell Viability Assay

**Figure 3:**
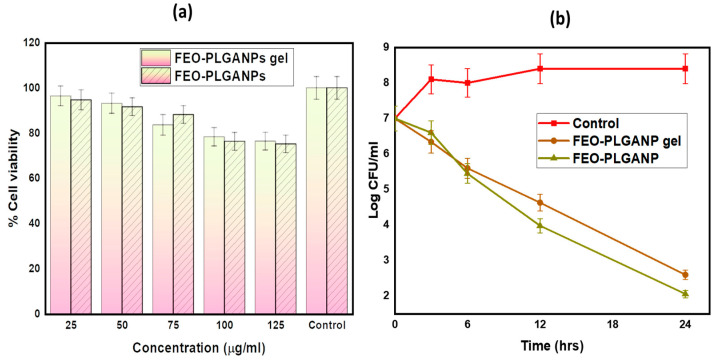
(**a**) Comparative in vitro cell viability assay of FEO–PLGANPs gel and FEO–PLGANPs; (**b**) Time-kill assay of the control, FEO–PLGANPs gel and FEO–PLGANPs.

Normal cell lines L929 were selected to determine the cytotoxicity of FEO–PLGANPs and FEO–PLGANPs gel, as shown in Figure 3a. According to the results, over 75% of the cells were alive after being cultured in either FEO-PLGANPs or FEO-PLGANPs gels. These results show that the cells can grow well and that the new synthetic nanoformulation has no adverse effects. These results also show low toxicity and excellent biocompatibility of FEO–PLGANPs and FEO–PLGANPs gel, indicating high potential for in vitro applications.

#### 2.2.4. In Vitro Antibacterial Activity

The antibacterial activities of pure FEO, FEO–PLGANPs and FEO–PLGANPs gel against *S. aureus* (MTCC 10787) were compared, as shown in Table 2**.** This might be related to the synergistic activity of PLGANPs and FEO. The antimicrobial activity of the FEO–PLGANPs gel was much higher than that of pure FEO. The difference in antimicrobial activity of pure FEO and FEO–PLGANPs against *S. aureus* was statistically significant (*p* < 0.05). The ability of FEO, FEO–PLGANPs and FEO–PLGANPs topical gels to increase antibacterial activity against S. aureus (MTCC 10787) was attributed to several factors, such as occlusive properties, specific drug-carrier interactions and close contact with the layers due to their small size. The FEO–PLGANPs and the FEO–PLGANPs gel showed almost the same value with MICs of 3.00 and 3.12 µg/mL, respectively. The naked FEO showed an MIC of 12.5 µg/mL. The FEO-PLGANPs and FEO-PLGANPs gel developed have been shown to be effective against *S. aureus* while being safe for the environment and having the potential to be used in skin care products.

#### 2.2.5. Time-Kill Assays

Figure 3b presents the rates of microbial killing by FEO–PLGANPs gel and FEO–PLGANPs when exposed to *S. aureus* (MTCC 10787) bacteria at three times MIC of respective therapies over a 24 h incubation period at 37 °C. FEO–PLGANPs gel and FEO–PLGANPs formulation demonstrated a rapid bactericidal effect, with a log reduction within a few hours.

#### 2.2.6. Stability Study

Long-term stability of drugs is of critical importance and can be difficult to achieve, especially in liquid formulations. Table 3 shows the stability study of FEO–PLGANPs gel. It was anticipated that the encapsulating of FEO in PLGANPs gel would lessen the EO’s instability caused by factors such as heat, air, and light. According to the results of our research, significant amounts of oil components are lost through decomposition and volatility.

## 3. Conclusions

*S. aureus* is an important zoonotic bacterium that is dangerous to human and livestock health throughout the world. *S. aureus* is resistant to several first-line antibiotics and multiple strategies are required to effectively control antibiotic-resistant behavior. Features such as biofilm formation, voluntary survival of intracellular organisms and increase in *S. aureus* resistance pose major challenges to its use in therapy, although the “pioneer method” of seeking new treatments is a logical approach needed to combat this disease. To overcome these challenges, it is very important to replace existing therapies with herbs based on fennel essential oils, which have excellent pharmacological profiles. Nanoparticles are considered as a promising tool for overcoming the problems in the treatment of infections caused by *S. aureus*. Compared to several first-line antibiotics, the obtained nanostructure is more effective in preventing *S. aureus* and has a greater ability to remove bacteria. Based on traditional herbal natural chemical compounds, we have shown that FEOs have a significant effect on the tested bacteria.

## 4. Materials and Methods

Fennel essential oils were purchased from Winlab Limited, Maharashtra, India. PLGA, PVA, carbopol, ethyl acetate and triethanolamine were purchased from Sigma-Aldrich, MO, USA. Double distilled water (DD) was used in all procedures. Numerous different chemicals and solvents of analytical grade were used without being altered in any way.

### 4.1. Methods

#### Preparation of Nanoparticles

First, we synthesized the FEO–PLGANPs using the solvent emulsion diffusion method, which we adopted with minor modifications from a previous publication [35]. A saturated aqueous phase and an organic phase were developed. A maximum of 200 mg PLGA was mixed in a volume of 10 ml ethyl acetate saturated with distilled water. Then, FEO (200 mg) was added to the organic phase. For this purpose, PVA (1% *w/v*) was dissolved in saturated water at 50 °C for 2 h. Then, the organic phase was added to the aqueous phase from top to bottom in an ice-cold bath and emulsified continuously for 15 min using an ultrasonic homogenizer. The oil-in-water (O/W) emulsion formed at room temperature. After completion of the nanosuspension, it was placed in an airtight container, frozen, freeze-dried and stored at 4 °C. The experiments were performed in triplicate.

### 4.2. Physicochemical Characterization of Nanoparticles

#### 4.2.1. Morphology of Nanoparticles

In order to investigate the architecture and morphology of the FEO–PLGANP, field emission electron microscopes (FESEM; JEOL JSM -6490LV) were applied to analyze the data. The samples were prepared for this by mounting them on copper tubes using double-sided adhesives. After that, each sample was evaluated using a FESEM with gold at a current of 20 mA for a duration of 120 s. The voltage used was 50 kV. For morphological research, HR-TEM (JEOL 2100 HRTEM, Seoul, Korea) was employed. To achieve this goal, a portion of the sample was cast onto a carbon copper mesh. It was then allowed to dry naturally at room temperature before being dyed with 2% uranyl acetate [36,37].

#### 4.2.2. Particle Size, Polydispersity Index and Surface Charge of Nanoparticles

After the dispersion had been diluted with deionized water to the necessary volume, the particle size (PS), polydispersity index (PDI), and zeta potential (ZP) of FEO-PLGANPs were measured at 25 °C using a zeta sizer (Nano ZS, Malvern Instruments Corp, Worcestershire, UK). Each measurement was performed three times [38].

#### 4.2.3. Drug Entrapment Efficiency (DEE%) of Nanoparticles

The DEE% of prepared FEO–PLGANPs was determined by the ultrafiltration method as per the method described in the literature [36,39]. The formulations (2 mL) were placed in ultracentrifuge filter tubes and centrifuged at 3000 rpm and 25 °C for 20–30 min. Centrifugal force forced the unconfined drugs through the centrifuge tubes. The unconfined FEO was determined by the UV method. The DEE % value was calculated using the following equation:

(1) $$\%DEE=\frac{\left(Total\;amount\;of\;FEO-Amount\;of\;FEO\;in\;supernatent\right)}{Total\;amount\;of\;FEO}\times 100$$

### 4.3. Preparation and Characterization of Hydrogel

#### Preparation of FEO–PLGANPs Gel

Basically, 0.5% *w*/*w* Carbopol was dispersed in distilled water with continuous magnetic stirring till dispersion was completed [40]. Under moderate, constant homogenization, FEO NPs (20% *w*/*w*) were mixed into a Carbopol dispersion and neutralized with triethanolamine (pH 6–7). The same procedure was used to prepare a gel blank formulation (control) to which neither oil nor Na nanoparticles were added. Both formulations were prepared in triplicate and analyzed in detail.

### 4.4. Characterization of FEO–PLGANPs Gel

#### 4.4.1. Rheological Study of FEO–PLGANPs Gel

Viscosity: Before each experiment, FEO–PLGANPs gels were kept at room temperature. The Brookfield viscosity monitor was used to test viscosity. The gel formulation is processed at modest speeds without damaging the gel structure. The viscosity was determined using the viscometer monitor.

pH: After weighing 0.5 g of gel and dissolving it in 50 mL of distilled water, the pH of the gel was determined.

Spreadability: The 20 × 20 cm horizontal plates were used to press the 0.5 g gel. Then a weight of 500 g was placed on the top plate and left there for about five minutes. The diameter of the dispersion circle is given in centimeters. The results are an average value obtained by combining the results of three different tests.

#### 4.4.2. Drug Release Studies of FEO–PLGANPs Gel

The in vitro drug release study of FEO from FEO-PLGANPs gel was performed at 37 °C using the dialysis bag technique in PBS with a pH of 6.8. Both free FEO and FEO–PLGANPs gel were in a dialysis bag. In a shaking incubator, the premix and the remaining tube filled with free oil were dialyzed against 40 mL of PBS at 37 ± 0.5 °C. A 3-mL sample was then collected at a fixed interval. Immediately after the PBS was removed, a sufficient quantity of new PBS was added to keep the sink in good working order. The amount of FEO released from the PLGANPs gel and FEO was measured by UV–vis spectrophotometry at a wavelength of 260 nm. Triplicate studies were performed.

#### 4.4.3. Drug Delivery Kinetics

We performed the kinetic study after the dissolution study. To find the most appropriate mathematical model for each formulation, DDSolver software was used to apply the zero-order, Higuchi, Korsmeyer-Peppas, Hixson and first-order kinetic models to the release profiles of the formulations [41]. When analyzing multiple variables in a model, it is advisable to interpret the R^2^ values. Since more variables can always be added to the model, the value can always be increased. This problem can be solved by calculating the adjusted R^2^ coefficient. The best model is selected from the adjusted R^2^ value obtained in each linear regression analysis.

#### 4.4.4. In Vitro Cell Viability Assay

To conduct the MTT experiment, we procured Normal Cell Line-L929 from the National Centre for Cell Sciences (NCCS), Pune. We performed the assay as per the method published by [39,42]. The medium in which the cells were grown was called Dulbecco’s modified Eagle’s medium (DMEM) and contained 10% fetal bovine serum (FBS). Cells were maintained at 37 °C in a humidified incubator containing 5% carbon dioxide.

Cell survival was determined using the MTT assay. Briefly, in this experiment, 1 × 10^4^ cancer cells in 100 µL of suspension were maintained in each well of 96-well plates. Following an initial incubation period of 24 h, cells were subjected to treatment with varying doses of FEO-PLGANPs gel and FEO-PLGANPs for an additional 24 h. Then, 20 µL of a working MTT solution at 5 mg/mL was added to each culture well. This was followed by an incubation period of 4 h. Absorbance (A) of each well was measured at 540 nm using a microculture plate reader [43].

(2) $$\%Cell\;viability=\frac{\left(A540\;nm\;treated\;cells\right)}{\left(A540\;nm\;untreated\;cells\right)}\times 100$$

#### 4.4.5. In Vitro Antibacterial Activity

Bacterial *S. aureus* (MTCC 10787) was prepared by adjusting the turbidity to 0.5 McFarland. Further dilution of the 0.5-McFarland suspension was performed at a ratio of 1:10 with sterilized nutrient broth and Sabouraud dextrose broth to obtain an inoculum of 108 CFU/mL according to the method described by Yousef and Danial, 2012 [44]. Two fold serial dilution broth microdilution was used to determine the MIC of FEO, FEO-PLGANPs, and FEO-PLGANPs gel against the aforementioned *S. aureus* strain. A sterile microtiter plate was used for the determination of MIC. Briefly, FEO, FEO–PLGANP and FEO–PLGANP gel stock solutions were prepared in water, ensuring complete solubility of 1 mg/mL. From well 1 to well 10, 100 µL each of nutrient broth and dextrose broth were added. 100 µL of a gel of FEO, FEO-PLGANP and FEO-PLGANP were added to the first well. The solution was successively diluted, starting with well 1 and ending with well 10. At each dilution interval, beginning with well 1 and ending with well 10, 100 µL of bacterial suspension was added. Overnight, 100 µL of the bacterial suspension was added to well 11, along with 100 µL of sterile broth as a growth control and 200 µL of sterile water. In well 12, the nutrient broth and the Sabouraud dextrose broth were used as sterility control and negative control, respectively. The plate was kept in an incubator at 37 °C for a full day. After incubation, the absorbance was measured using an ELISA reader (Erba) operating at a wavelength of 640 nm. The procedure described above was applied to each microorganism species. Sample and standard concentrations were determined that would inhibit the growth of the bacteria by 50%. The test was performed in three times to minimize human error.

#### 4.4.6. Time-Kill Kinetics Assay

Time-kill kinetics of FEO–PLGANP gel was carried out following the procedure described by [45]. Concentrations were prepared that were three times the MIC of the FEO–PLGANP gel. Sterile water was added to this bacterial broth, and the test samples were added as a negative control. The viability of the bacterial cells was monitored continuously for 24 h. Samples were collected at specific time intervals and serially diluted in PBS. They were incubated overnight at 37 °C. At the end of incubation, the growth of bacteria in the colonies on the plates was counted. The colony-forming unit (CFU) was calculated and converted to log CFU/mL, and the time was plotted on a graph.

#### 4.4.7. Stability Study

The effect of temperature on the FEO–PLGANP gel was further investigated using short-term changes in physical stability. The formulations of the FEO–PLGANP gels were kept at ambient temperature for 0, 15 and 30 days according to the procedures established by the International Conference on Harmonization, and the samples were tested for their physicochemical properties at each of these intervals [46]. All visible changes were analyzed, including precipitation, turbidity, crystallization, color, particle size, PDI and ZP.

## Figures and Tables

**Table 1:** Drug-release kinetics of different formulations.

Formulation	Zero Order	Higuchi	First Order	Kors–Peppas	Hixson
Bare FEO	0.9145	0.9839	0.9239	0.9002	0.9506
FEO–PLGANPs	0.996	0.9117	0.9882	0.6994	0.9925
FEO–PLGANPs gels	0.9905	0.8827	0.9781	0.6591	0.9834

**Table 2:** Comparative in vitro antibacterial activity of FEO–PLGANPs gel, FEO–PLGANPs, PLGANPs and FEO against *S. aureus* (MTCC 10787) bacteria.

Compound	MIC (µg/mL)
Vancomycin	4.92
FEO–PLGANPs gel	3.12
FEO–PLGANPs	3.00
PLGANPs	NA
FEO	12.5

**Table 3:** The effects of storage conditions on the physical and chemical properties of the formulation of FEO–PLGANPs gel at ambient temperature.

Days	Color	Texture	Consistency	pH	EE%
0	Off-white	smooth	consistent	6.0	66.4
15	Off-white	smooth	consistent	6.4	64.4
30	Off-white	smooth	consistent	6.7	62.05

## Data Availability

Not applicable.
